# Verbascoside restores gastrointestinal integrity and attenuates inflammation in a rat model of 5-FU-induced mucositis

**DOI:** 10.1007/s12032-025-02823-0

**Published:** 2025-06-16

**Authors:** Ugochukwu Chukwunyere, Serkan Sayıner, Merve Mercan, Şule Çetinel, İhsan Çaliş, Ahmet Özer Sehirli

**Affiliations:** 1 Department of Pharmacology, Faculty of Pharmacy, Near East University, 99138 Nicosia, North Cyprus; 2 Department of Biochemistry, Faculty of Veterinary Medicine, Near East University, 99138 Nicosia, North Cyprus; 3 Department of Pharmacology and Clinical Pharmacy, Faculty of Pharmacy, Girne American University, 99428 Kyrenia, North Cyprus; 4 Department of Histology and Embryology, School of Medicine, Marmara University, 34722 Istanbul, Turkey; 5 Department of Pharmacognosy, Faculty of Pharmacy, Near East University, 99138 Nicosia, North Cyprus; 6 Department of Pharmacology, Faculty of Dentistry, Near East University, 99138 Nicosia, North Cyprus

**Keywords:** Verbascoside, Acteoside, 5-fluorouracil, Mucositis, Cytokines, Oxidative stress

## Abstract

**Background:**

This study investigated the protective effects of verbascoside (VER) against 5-fluorouracil (5-FU)-induced gastrointestinal mucositis in Wistar albino rats.

**Methods and results:**

The study involved 30 female rats that were equally divided into five groups as follows: Control group, 5-FU group (400 mg/kg, IP), VER-only group (0.2 mg/kg, IP), 5-FU (400 mg/kg, IP) + VER (0.2 mg/kg, IP) group, and 5-FU (400 mg/kg, IP) + VER (0.4 mg/kg, IP) group. All animals were euthanized four days after 5-FU administration. Gastrointestinal tissues (esophagus, stomach, duodenum, jejunum, ileum, and colon) and blood sera were collected for histopathological and biochemical analyses. Tissue and sera analyses showed that 5-FU caused significant alterations marked by increases in matrix metalloproteinases (MMP-1, -2, -8), alkaline phosphatase (ALP), aspartate aminotransferase (AST), lactate dehydrogenase (LDH), tumor necrosis factor-alpha (TNF-α), and interleukin-1 beta (IL-1β) levels and decreases in tissue inhibitor of metalloproteinases-1 (TIMP-1), albumin, and total protein levels. VER treatment effectively attenuated these 5-FU-induced changes, with trends toward improved histological outcomes at higher doses.

**Conclusion:**

The findings strongly suggest that VER offers significant protection, and these results warrant further investigation into its potential clinical application as an adjunct therapy to mitigate gastrointestinal and other toxicities associated with 5-FU chemotherapy.

## Introduction

5-Fluorouracil (5-FU) can cause gastrointestinal mucositis, a debilitating side effect that reduces quality of life, interferes with anticancer therapy and increases healthcare costs [1, 2]. The pathogenesis of 5-FU-related mucositis has been described to include inflammation, gut barrier disruption, oxidative damage, and cellular apoptosis [3–5]. Stress has also been reported to significantly exacerbate inflammation in the gut by activating the gut-brain axis and disrupting the gut microbiota [6]. Consequently, some studies suggest that in addition to targeting the gut microbiota, probiotic supplements [7], as well as agents with antimicrobial [8], antioxidant [9], and anti-inflammatory [10] properties show promise in treating associated symptoms. Despite advances in mucositis research, a definitive treatment for chemotherapy-induced gastrointestinal mucositis remains elusive.

Numerous plant-derived substances have demonstrated the capacity to bolster organismal defenses against physiological and environmental stressors. Verbascoside (VER), also known as acteoside, a water-soluble phenylethanoid glycoside [11], is one such compound. VER is widely distributed in nature and has been identified in several medicinal plants that exert various pharmacological effects [12]. These effects are attributed to its ability to suppress pro-inflammatory mediators [13, 14], in addition to its antioxidative [15–18], antimicrobial [19, 20], and anticancer [21–24] activities.

Given that VER has a protective effect against stress-induced ulcers, and considering the well-established use of Wistar rats as a model for inflammation-induced tissue damage in humans [3, 25], this study takes a broader approach than most compartmentalized mucositis research, which typically targets either the oral or intestinal regions. Instead, the effects and underlying protective mechanisms of different doses of VER against 5-FU-induced mucositis are investigated throughout the gastrointestinal tract, from the esophagus to the colon. By examining both structural changes in gastrointestinal tissues and changes in biochemical parameters in serum, this study demonstrates the systemic nature of mucositis as it occurs in patients undergoing chemotherapy.

## Materials and methods

### Animals

The study utilized female albino Wistar rats (*n* = 30) aged 12 weeks and weighing 250–300 g. These animals were obtained from the institution’s faculty of veterinary medicine and housed under controlled conditions (22 ± 0.5 °C, 12-h light/dark cycle) in pathogen-free polypropylene cages, with ad libitum access to food and water. All research protocols were performed in line with the principles of the Declaration of Helsinki and approved by the Near East University Animal Ethics Committee (Protocol No: 2021/136-137).

### Drug and extracts

5-FU used in this study was obtained from Kocak Farma (Istanbul, Turkey). The pre-isolated and characterized VER was sourced from the pharmacognosy department, following their prior work on *Phlomis* L*.* species [26]. VER was dissolved in distilled water, and all drugs and reagents were freshly prepared before administration.

### Induction of intestinal mucositis

This study utilized an established model of gastrointestinal mucositis in albino Wistar rats, as detailed in prior publication [27]. To induce mucositis, animals in the mucositis group were fasted overnight and then received a single intraperitoneal injection of 5-fluorouracil at a dose of 400 mg/kg on day 1.

### Experimental design

The study employed a five-group design with six rats (*n* = 6) per group. The control group received daily intraperitoneal injections (IP) of saline for four days. The 5-FU group received a single intraperitoneal injection of 5-FU (400 mg/kg) on day 1, followed by intraperitoneal injections of saline for the subsequent four days. The VER-only group received daily intraperitoneal injections of VER at 0.2 mg/kg for four days. The remaining two groups received 5-FU (400 mg/kg, IP) on day 1, followed by daily VER treatment for four days at either 0.2 mg/kg or 0.4 mg/kg (IP). On day 5, all rats were euthanized via intraperitoneal injection with a mixture of ketamine (70 mg/kg) and xylazine (10 mg/kg). Blood samples were collected via cardiac puncture into serum separator tubes, and the following tissues were excised: esophagus, stomach, duodenum, jejunum, ileum, and colon.

### Biochemical assays

Analyses were performed at the Veterinary Hospital Diagnostic Laboratory of Near East University. Blood samples were centrifuged (3000 rpm, 10 min) and stored at − 80 °C until analysis. Serum levels of tumor necrosis factor-alpha (TNF-α), interleukin-1 beta (IL-1β), matrix metalloproteinases (MMP-1, MMP-2, MMP-8), and tissue inhibitor of metalloproteinases-1 (TIMP-1) were quantified using commercially available rat-specific ELISA kits (ELR-TNFα and ELR-IL1β, RaybioTech Inc., GA, USA) according to the manufacturer's instructions. Automated washing steps were performed with a Mindray MW-12A Microplate Washer (Shenzhen, China), and absorbance was measured at 450 nm with a Mindray MR-96A Microplate Reader (Shenzhen, China).

Serum malondialdehyde (MDA), an indicator of lipid peroxidation, was quantified according to a previously described method [28], which involves the reaction of MDA with thiobarbituric acid (TBA) under acidic conditions at 100 °C. The resulting absorbance was read at 530–540 nm using a Molecular Devices VersaMax Tunable Microplate Reader (CA, USA).

Serum activities of lactate dehydrogenase (LDH), alanine transaminase (ALT), aspartate aminotransferase (AST), and alkaline phosphatase (ALP), which are biomarkers of cellular leakage indicative of hepatocellular damage, were measured using a Mindray BS240-Vet automated analyzer (Shenzhen, China).

### Histopathological and morphometric analysis

Following euthanasia, tissue segments were collected and fixed in 10% formaldehyde for subsequent histopathological and morphometric evaluations, as previously outlined [29]. These fixed tissues were then paraffin-embedded, sectioned into 5 μm -thick slices, and stained with hematoxylin and eosin for microscopic examination. The histopathological analyses were conducted by an experienced histopathologist to assess the severity of mucositis using previously established scoring system [30, 31]. The standardized histological scoring system was employed to assess the severity of gastrointestinal mucositis in each segment (esophagus, stomach, duodenum, jejunum, ileum, and colon). This system assigned scores ranging from 0 (normal) to 3 (severe) based on pre-defined criteria.

Edema was evaluated as:

- 3 (severe): fluid accumulation both externally and internally within the intestinal wall.
- 2 (moderate): edema confined to the mucosa.
- 1 (mild): intermediate between normal and moderate.
- 0 (normal): no edema observed.

Hemorrhage intensity was graded as:

- 3 (severe): clot and intraluminal blood present.
- 2 (moderate): mucosal and wall hematomas present.
- 1 (mild): vessel dilation observed.
- 0 (normal): no hemorrhage detected.

Histological and biochemical analyses were integrated to determine the effects of VER treatment on gastrointestinal mucositis.

### Statistical analysis

Data were analyzed using GraphPad Prism version 7.0 (GraphPad Software Inc., San Diego, CA, USA). Normally distributed data were analyzed by one-way ANOVA followed by Tukey’s post hoc test. Non-parametric data were analyzed using the Kruskal–Wallis test with Dunn’s post hoc test. Data are presented as mean ± standard deviation (SD), and statistical significance was defined as p < 0.05.

## Results

**Fig. 1:**
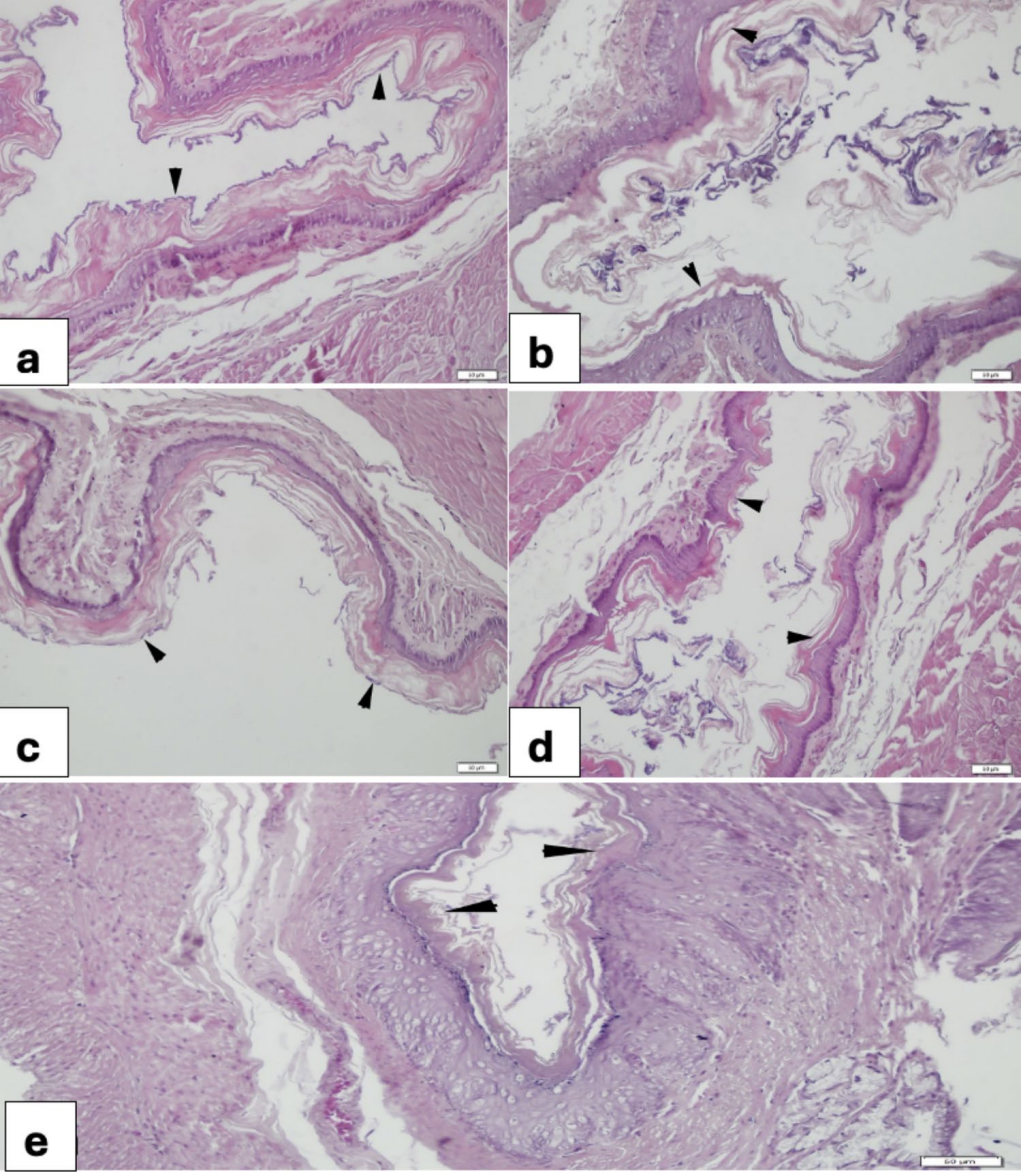
Morphological changes in the esophagus **a** Control group, regular layout of keratin layer (arrowheads) and epithelium; **b** 5-FU 400 mg/kg group, severe detachment of keratin layer from epithelium (arrowheads); **c** VER 0.2 mg/kg group, regular layout of keratin and epithelium (arrowheads); **d** 5-FU + VER 0.2 mg/kg group, regional attachment of keratin (arrowheads); **e** 5-FU + VER 0.4 mg/kg group, regular attachment of keratin layer to epithelium (arrowheads)

Histopathological and Morphometric results:

Esophagus:

Esophageal examination revealed severe degeneration with desquamation of the keratin and epithelial layers in the 5-FU group (Fig. 1b) compared to the control group, which showed a well-organized mucosa with intact keratin and epithelium (Fig. 1a). VER (0.2 mg/kg) group (Fig. 1c) had similar pattern as that of the control group. The 5-FU + VER (0.2 mg/kg) group showed detachment of keratin layer in some regions (Fig. 1d) but 5-FU + VER (0.4 mg/kg) group had a better attachment of keratin layer to epithelium (Fig. 1e).

**Fig. 2:**
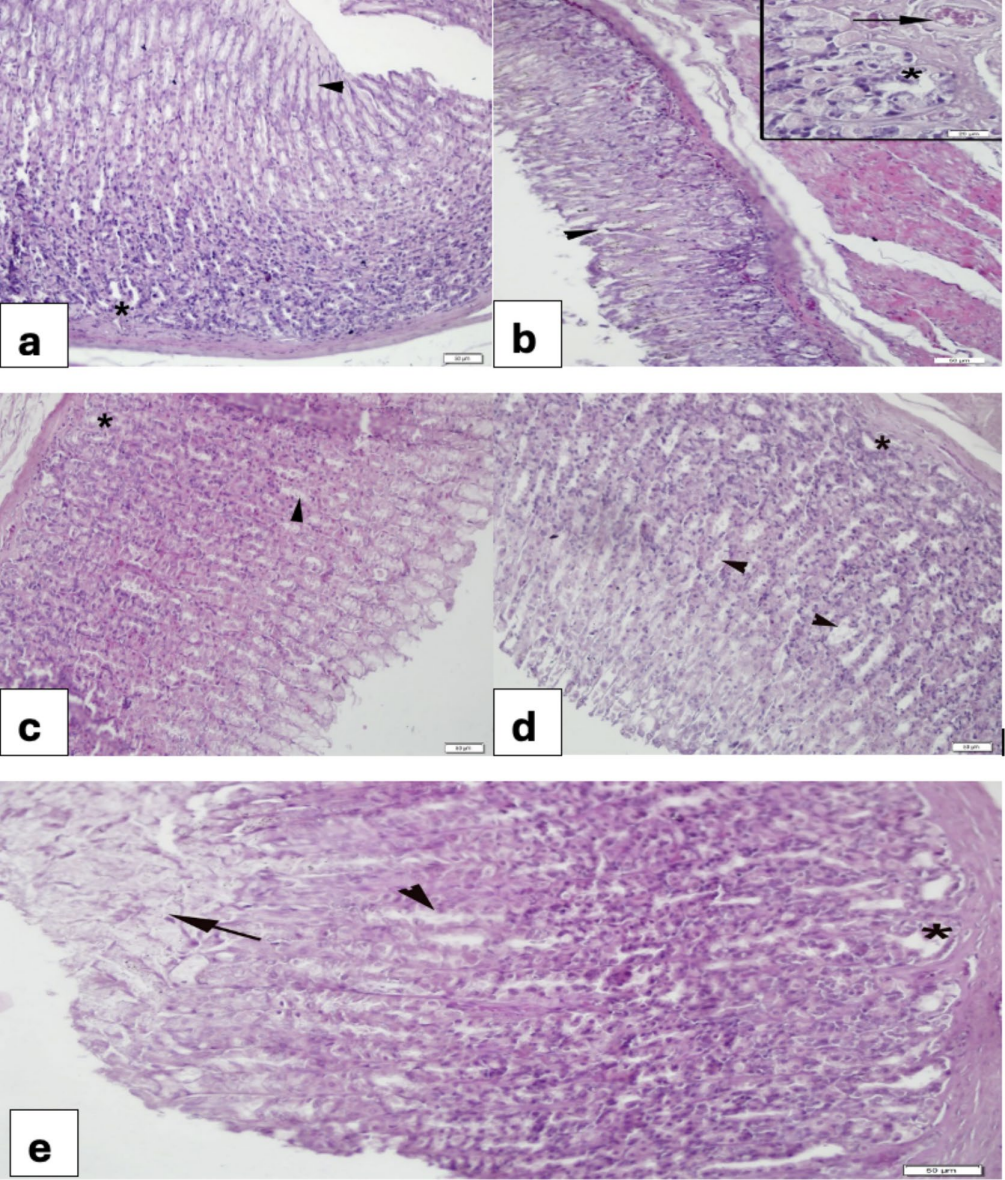
Morphological changes in the stomach **a** Control group, regular layout of gastric pits (arrowheads) and glands (*); **b** 5-FU 400 mg/kg group, severe degeneration of both gastric pits (arrowhead) and glands (*), congestion (arrow); **c** VER 0.2 mg/kg group, regular appearance of both gastric pits (arrowhead) and glands (*); **d** 5-FU + VER 0.2 mg/kg group, moderate dilation in gastric pits (arrows) and glands (*); **e** 5-FU + VER 0.4 mg/kg group, reduced dilation of gastric pits (arrow) and mild dilation in glands (*), note the prominent mucous collection on the mucosa (arrow)

Stomach:

Examination of the stomach revealed a normal organization with evident gastric pits and glands in the control group (Fig. 2a). In contrast, the 5-FU group displayed dilated glands and gastric pits along with congestion (Fig. 2b). VER (0.2 mg/kg) group (Fig. 2c) had similar appearance to that of the control group. 5-FU + VER (0.2 mg/kg) group gastric pits showed dilation in some areas (Fig. 2d). In the 5-FU + VER (0.4 mg/kg) group, dilation of both gastric pits and glands reduced but bizarre mucous was prominent (Fig. 2e).

### Duodenum

**Fig. 3:**
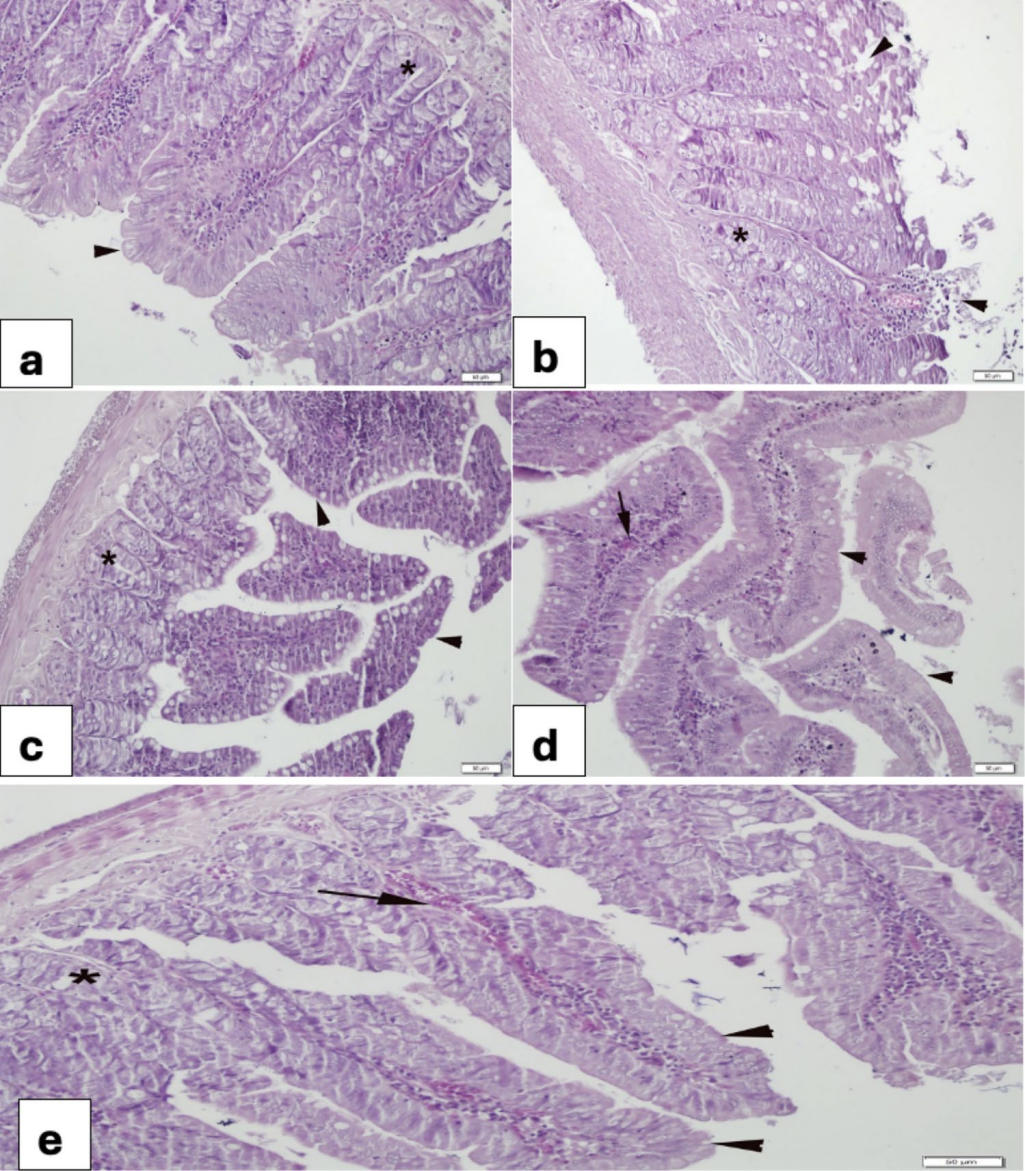
Morphological changes in the duodenum **a** Control group, regular layout of the epithelium of villi (arrowhead) and glands (*); **b** 5-FU 400 mg/kg group, severe desquamation of the villi (arrowheads) degeneration of the glands (*); **c** VER 0.2 mg/kg group, regular villi epithelium (arrowheads) and glands (*); **d** 5-FU + VER 0.2 mg/kg group, nearly re-epithelized villi (arrowheads) and congestion (arrow); **e** 5-FU + VER 0.4 mg/kg group, completed re-epithelization (arrowheads), congestion (arrow), glands (*)

The duodenum of the control group showed a regular epithelium and glands (Fig. 3a). In the 5-FU group, a bizarre degeneration of the epithelium and glands as well as congestion was observed (Fig. 3b). In the VER (0.2 mg/kg) group (Fig. 3c), a normal structure was observed. In the 5-FU + VER (0.2 mg/kg) group, re-epithelialization of the villi occurred in addition to congestion (Fig. 3d). In the 5-FU + VER (0.4 mg/kg) group, re-epithelialization was complete (Fig. 3e).

### Jejunum

**Fig. 4:**
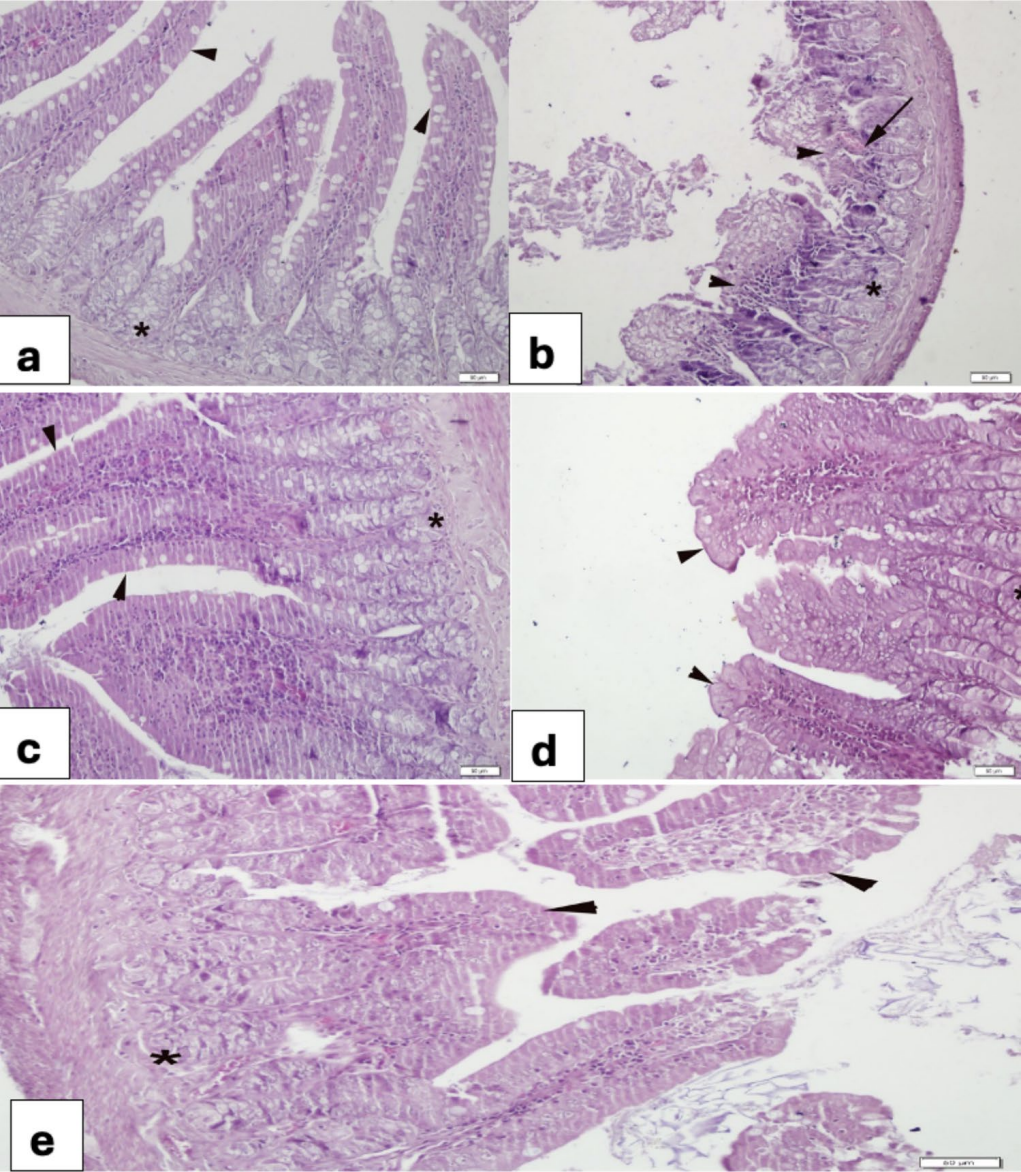
Morphological changes in the jejunum **a** Control group, regular layout of villi epithelium (arrowheads) and glands (*); **b** 5-FU 400 mg/kg group, more than half of the villi desquamation (arrowheads), gland (*); **c** VER 0.2 mg/kg group, regular morphology of villi epithelium (arrowheads) and gland (*); **d** 5-FU + VER 0.2 mg/kg group, nearly completed re-epithelization (arrowheads); **e** 5-FU + VER 0.4 mg/kg group, prominent epithelization of the villi (arrowheads)

The control jejunum showed a regular villous epithelium rich in goblet cells and a regular glandular morphology (Fig. 4a). In the 5-FU group, severe desquamation of the villi and glands were observed (Fig. 4b). In the VER (0.2 mg/kg) group (Fig. 4c) normal villous epithelium and glands was observed. the 5-FU + VER (0.2 mg/kg) group showed partial epithelialization (Fig. 4d), while the 5-FU + VER (0.4 mg/kg) group showed marked epithelialization (Fig. 4e).

### Ileum

**Fig. 5:**
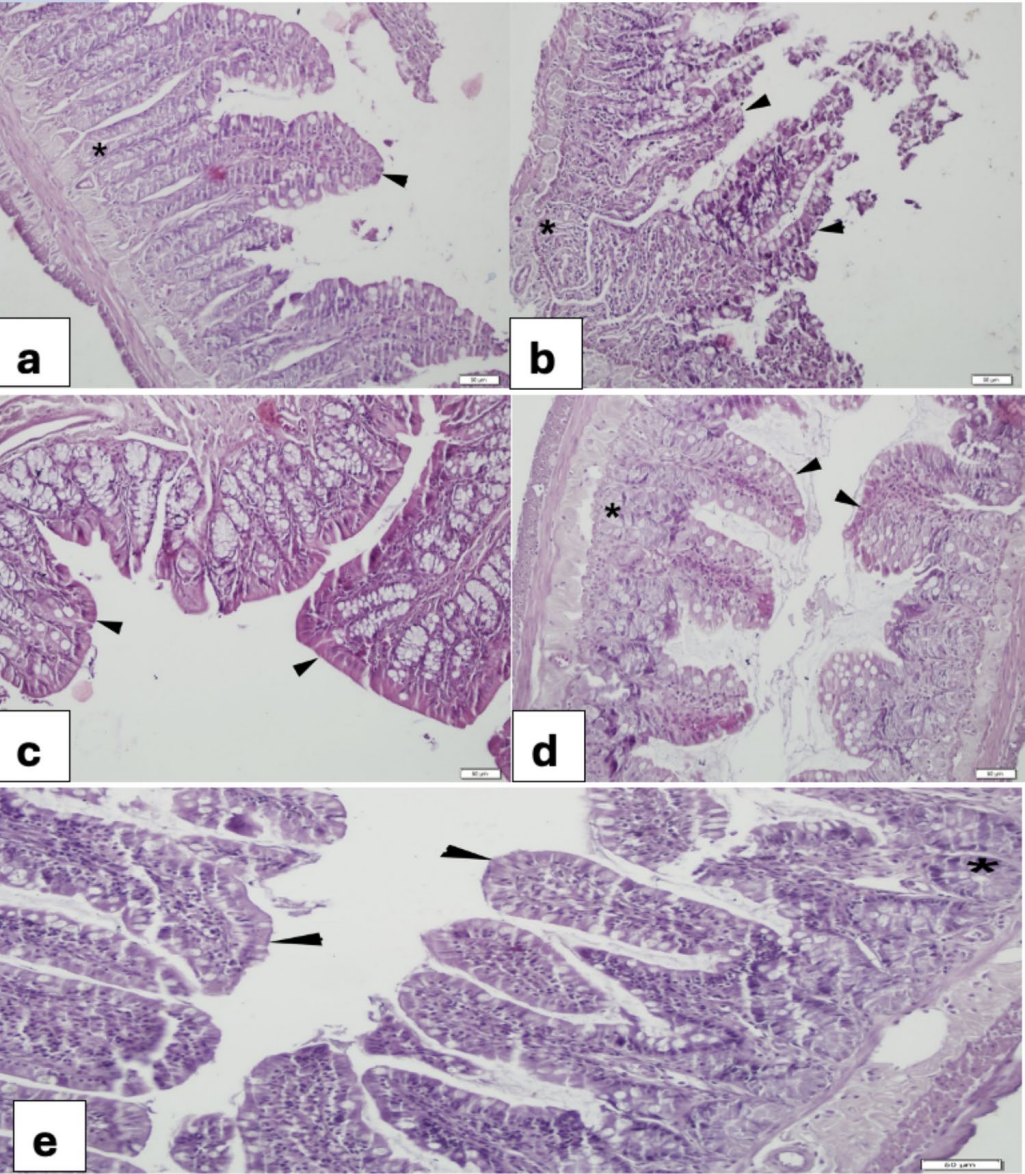
Morphological changes in the ileum **a** Control group, regular epithelium (arrowheads) and glands (*); **b** 5-FU 400 mg/kg group, severely detached villi (arrowheads) and degenerated glands (*); **c** VER 0.2 mg/kg group, regular structures of both villi (arrowheads) and glands (*); **d** 5-FU + VER 0.2 mg/kg group, partially re-epithelized villi (arrowheads) and gland (*); **e** 5-FU + VER 0.4 mg/kg group, nearly full epithelization of the villi (arrowheads), glands(*)

The control ileum showed a regular epithelium and glands with goblet cells (Fig. 5a), whereas in the 5-FU group, the villus structure was desquamated by more than half and the glands were degenerated (Fig. 5b). In the VER (0.2 mg/kg) group (Fig. 5c), the epithelium of the villi retained its regularity. In the 5-FU + VER (0.2 mg/kg) group (Fig. 5d), the epithelial structure was mostly regular, while the 5-FU + VER (0.4 mg/kg) group (Fig. 5e) showed almost complete epithelial regularity.

### Colon

**Fig. 6:**
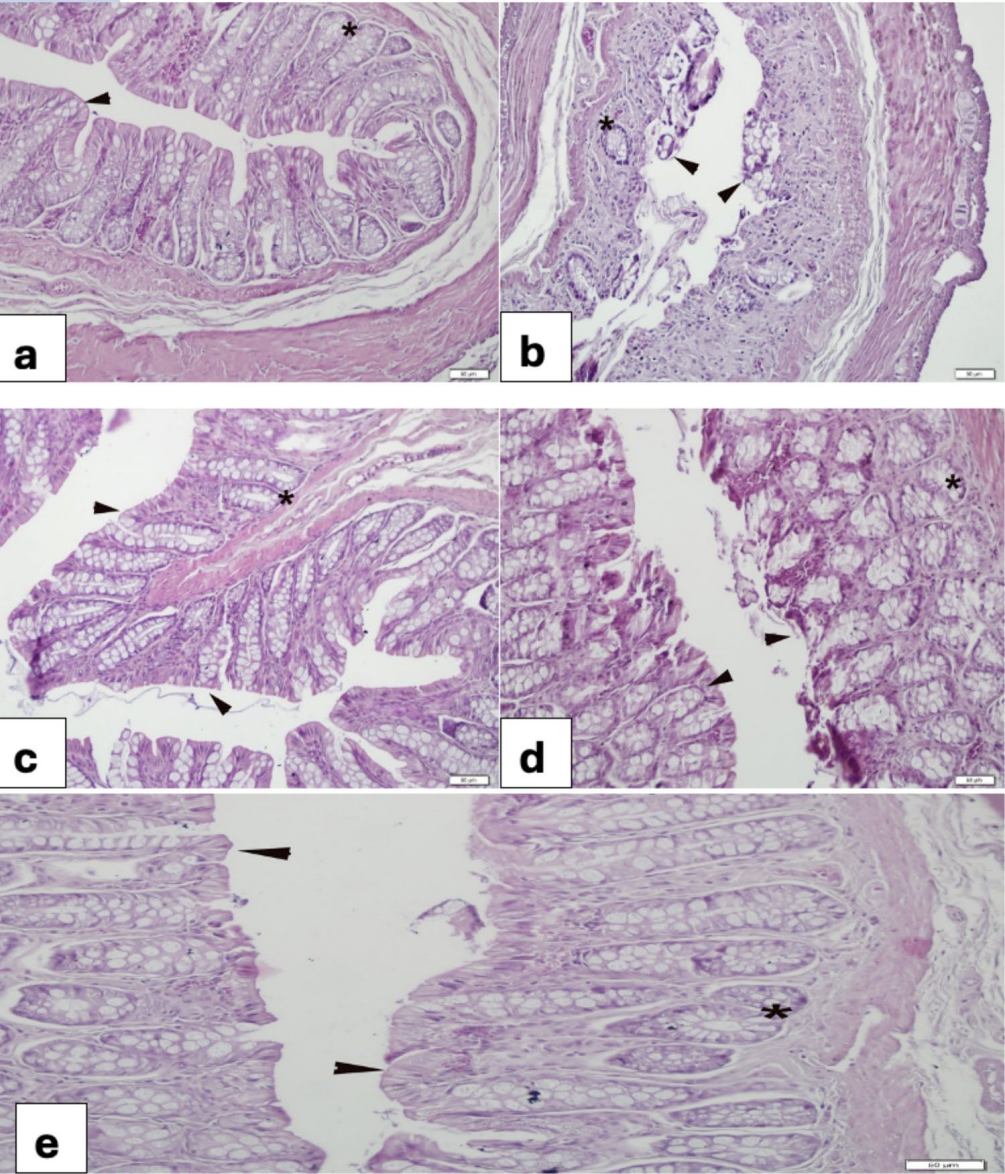
Morphological changes in the colon **a** Control group, regular layout of epithelium (arrow) and gland (*); **b** 5-FU 400 mg/kg group, severe desquamation of the epithelium (arrowheads) hypertrophy of glands (*); **c** VER 0.2 mg/kg group, regular structure of epithelium (arrowheads) and glands (*); **d** 5-FU + VER 0.2 mg/kg group, regeneration at the tip (arrowheads); **e** 5-FU + Verbo 0.4 mg/kg group, complete re-epithelization (arrowheads)

The control colon showed a regular structure with intact epithelium and crypts (Fig. 6a). In the 5-FU group, severe desquamation of the epithelium and crypt degeneration were observed (Fig. 6b). The VER (0.2 mg/kg) group showed a normal structure (Fig. 6c). In the 5-FU + VER (0.2 mg/kg) group, the tips of the tissue were partially desquamated (Fig. 6d), while in the 5-FU + VER (0.4 mg/kg) group, epithelialization was nearly complete (Fig. 6e).

Effects of 5-Fluorouracil and Verbascoside on Serum Malondialdehyde Levels.

Rats treated with 5-FU (400 mg/kg) + saline had a significant increase in MDA levels compared to the control group (p < 0.0001). Notably, VER treatment (0.2 and 0.4 mg/kg) significantly attenuated the 5-FU-induced increase in MDA levels (p < 0.001, respectively), suggesting a possible protective effect against oxidative stress (Table 1).

**Table 1:** Effects of 5-fluorouracil and verbascoside on serum malondialdehyde (MDA) levels

	Control	VER	5-FU	5-FU + VER 0.2 mg/kg	5-FU + VER 0.4 mg/kg
MDA (µmol/L)	6.62 ± 0.90	7.42 ± 1.12^**++++**^	50.35 ± 11.42^****^	15.85 ± 2.83^**+++**^	16.86 ± 3.13^**+++**^

^****^p < 0.0001 compared with the Control group; ^+++^*p* < 0.001, ^++++^*p* < 0.0001 compared with the 5-FU group. Data are expressed as mean ± SEM (*n* = 6)

Effects of 5-Fluorouracil and Verbascoside on Tumor Necrosis Factor-alpha and Interleukin-1 beta Levels.

Previous studies have shown that 5-FU can trigger inflammatory responses that exacerbate tissue damage, thereby amplifying its overall adverse effects. In this study, 5-FU significantly increased the serum level of pro-inflammatory cytokines- TNF-α and IL-1β, compared to the control group (*p* < 0.0001 and *p* < 0.01, respectively). VER treatment, however, effectively attenuated these 5-FU-induced inflammatory responses, with both doses of VER (0.2 and 0.4 mg/kg) significantly reducing TNF-α and IL-1β levels (*p* < 0.0001 and *p* < 0.01, respectively) (Table 2).

**Table 2:** Effects of 5-fluorouracil and verbascoside on tumor necrosis factor-alpha (TNF-α), and Interleukin-1 beta (IL-1 β) Levels

	Control	VER	5-FU	5-FU + VER 0.2 mg/kg	5-FU + VER 0.4 mg/kg
TNF- α (pg/mL)	8.84 ± 1.51	19.78 ± 3.61^**++++**^	134.80 ± 68.20^****^	20.26 ± 5.13^**++++**^	35.08 ± 7.87^**++++**^
IL-1 β (pg/mL)	153.90 ± 22.85	145.10 ± 20.92^**+++**^	365.60 ± 70.98^**^	184.40 ± 21.36^**++**^	155.40 ± 17.78^**++**^

^**^p < 0.01, ***p < 0.001 compared with the Control group; ^++^*p* < 0.01, ^+++^*p* < 0.001, ^++++^*p* < 0.0001 compared with the 5-FU group. Data are expressed as mean ± SEM (*n* = 6)

Effects of 5-Fluorouracil and Verbascoside on Serum Matrix Metalloproteinases and Tissue Inhibitor of Metalloproteinases-1 Expression.

5-FU administration significantly increased the expression of serum MMPs (-1, -2, and -8). This elevated MMPs (-1, -2, and -8) expression induced by 5-FU indicate excessive extracellular matrix degradation, which is often associated with tissue damage and inflammation.

MMP-1 expression was significantly upregulated in the 5-FU group compared to the control (*p* < 0.0001). VER treatment reversed this 5-FU-induced increase in serum MMP-1 expression, with a more significant effect observed in the 5-FU + VER (0.4 mg/kg) group (*p* < 0.05) (Table 3), suggesting that higher doses of VER may provide greater attenuation of MMP-1 expression.

**Table 3:** Effects of 5-fluorouracil and verbascoside on serum matrix metalloproteinases (MMPs) and tissue inhibitor of metalloproteinases-1 (TIMP-1) expression

	Control	VER	5-FU	5-FU + VER 0.2 mg/kg	5-FU + VER 0.4 mg/kg
MMP-1 (pg/mL)	0.83 ± 0.18	1.66 ± 0.32^**++**^	3.97 ± 0.60^****^	2.60 ± 0.40	1.91 ± 0.36^**+**^
MMP-2 (pg/mL)	20.60 ± 1.53	22.60 ± 1.52^**++++**^	54.30 ± 6.34^****^	25.37 ± 2.12^**++++**^	29.88 ± 2.73^**+++**^
MMP-8 (pg/mL)	85.34 ± 5.90	86.10 ± 10.67^**++++**^	195.80 ± 19.20^****^	119.60 ± 16.24^**++**^	101.60 ± 7.04^**+++**^
TIMP-1 (pg/mL)	2497 ± 226.00	2811 ± 262.40^**++++**^	584.40 ± 209.90^****^	1759 ± 327.30^**+**^	1701 ± 182.80^**+**^

^****^p < 0.0001 compared with the Control group; ^**+**^*p* < 0.05, ^**++**^*p* < 0.01, ^**+++**^*p* < 0.001, ^**++++**^*p* < 0.0001 compared with the 5-FU group. Data are expressed as mean ± SEM (*n* = 6)

Similar to MMP-1, treatment with 5-FU significantly increased serum MMP-2 levels in the 5-FU group compared to the control group (*p* < 0.0001). However, both VER doses (0.2 mg/kg and 0.4 mg/kg) significantly attenuated this 5-FU-induced increase in MMP-2 (*p* < 0.0001 and *p* < 0.001, respectively) (Table 3).

As observed with MMP-1 and MMP-2, treatment with 5-FU significantly increased MMP-8 expression in the 5-FU group relative to the control group (*p* < 0.0001). VER treatment at both 0.2 mg/kg and 0.4 mg/kg significantly attenuated this 5-FU-induced increase in MMP-8 expression compared to the 5-FU group (*p* < 0.01 and *p* < 0.001, respectively). Notably, the expression of serum MMPs -1, -2, and -8 was significantly lower in the VER (0.2 mg/kg) only group compared to the 5-FU group (*p* < 0.01–0.0001), suggesting a potential baseline-lowering effect of VER (Table 3).

TIMP-1 expression significantly decreased in the 5-FU group compared to the control (*p* < 0.0001). TIMP-1 is a natural inhibitor of MMPs, so the decrease induced by 5-FU results in reduced control over MMP activity, which may exacerbate tissue damage. VER treatment reversed this effect, with significantly higher TIMP-1 levels observed in the 5-FU + VER (0.2 mg/kg) group and the 5-FU + VER (0.4 mg/kg) group compared to the 5-FU group (*p* < 0.05 for both) (Table 3).

Effects of 5-Fluorouracil and Verbascoside on Serum Albumin, Total Protein, and Creatinine Levels.

5-FU significantly reduced serum albumin and total protein levels compared to the control group (*p* < 0.01 and *p* < 0.05, respectively). While treatment with VER (0.2 mg/kg and 0.4 mg/kg) tended to increase albumin and total protein levels and decrease creatinine in all treated groups, these effects were not statistically significant (*p* > 0.05 for all comparisons) (Table 4).

**Table 4:** Effects of 5-fluorouracil and verbascoside on serum albumin, total protein. and creatinine levels

	Control	VER	5-FU	5-FU + VER 0.2 mg/kg	5-FU + VER 0.4 mg/kg
Albumin (g/dL)	4.60 ± 0.37	3.77 ± 0.17	2.61 ± 0.39^**^	4.30 ± 0.35	4.10 ± 0.22
Total proteins (g/dL)	6.96 ± 0.88	6.52 ± 1.23	4.75 ± 1.69^*^	5.83 ± 0.58	6.05 ± 1.12
Creatinine (mg/dl)	0.62 ± 0.07	0.69 ± 0.14	1.33 ± 0.27*	0.81 ± 0.11	0.96 ± 0.16

^*^p < 0.05, **p < 0.01 compared with the Control group. Data are expressed as mean ± SEM (*n* = 6)

Effects of 5-Fluorouracil and Verbascoside on Serum Enzyme Levels.

Compared to the control group, 5-FU (400 mg/kg) significantly increased ALP, AST, and LDH levels (*p* < 0.001, *p* < 0.05, and *p* < 0.0001, respectively), indicative of possible liver damage. VER treatment reversed these effects. Specifically, treatment with low dose VER (0.2 mg/kg) significantly decreased ALP levels compared to the 5-FU group (*p* < 0.001), demonstrating a greater effect than high dose VER (0.4 mg/kg) (*p* < 0.05). In contrast, treatment with the high-dose VER (0.4 mg/kg) significantly reduced the LDH levels compared to the 5-FU group (*p* < 0.05). Treatment with the various doses of VER had no significant (*p* > 0.05) effect on serum AST and ALT levels in any of the treated groups, as compared to the 5-FU group (Table 5).

**Table 5:** Effects of 5-fluorouracil and verbascoside on serum enzyme levels

	Control	VER	5-FU	5-FU + VER 0.2 mg/kg	5-FU + VER 0.4 mg/kg
ALT (U/L)	24.29 ± 5.06	22.59 ± 4.54	42.06 ± 8.78	24.95 ± 4.12	26.67 ± 4.03
ALP (U/L)	17.15 ± 2.99	37.29 ± 6.36^**+++**^	75.49 ± 9.22^***^	37.36 ± 6.26^**+++**^	47.36 ± 4.50^**+**^
AST (U/L)	92.38 ± 9.78	116.30 ± 8.19	141.70 ± 12.45^*^	105.50 ± 7.64	122.60 ± 11.65
LDH (U/L)	790.60 ± 96.29	1247 ± 172.80^**+++**^	3213 ± 511.00^****^	2142 ± 210.70	1919 ± 294.20^**+**^

^*^p < 0.05, ***p < 0.001, ****p < 0.0001 compared with the Control group; ^**+**^*p* < 0.05, ^**+++**^*p* < 0.001 compared with the 5-FU group. Data are expressed as mean ± SEM (*n* = 6)

## Discussion

This study investigated the protective effects of VER against 5-FU-induced gastrointestinal mucositis and explored the possible underlying mechanisms. The results expand the understanding of gastrointestinal mucosal protection and reduction of associated toxic damage in patients undergoing chemotherapy. The study also provides a solid basis for the clinical use of plant-derived compounds for the prevention of mucositis caused by chemotherapy.

Chemotherapeutic agents, such as 5-FU, are associated with elevated serum enzyme levels and LDH activity, as well as tissue damage mediated by oxidative stress and inflammatory cytokine production [3–5, 32]. As a result of these adverse effects on various tissues and organs, 5-FU is a common agent for inducing tissue damage in experimental studies [33, 34]. Considering the established VER anti-inflammatory [13, 14, 35, 36] and antioxidant properties [15–18, 37, 38], we hypothesized that VER could attenuate 5-FU-induced gastrointestinal damage and promote tissue regeneration. To test this hypothesis, we performed histological and biochemical analyses of gastrointestinal tissues and serum.

In this study, 5-FU significantly increased serum levels of ALP, AST, and LDH, indicating hepatic dysfunction that may contribute to systemic toxicity and disrupt various metabolic processes. Such enzyme alterations are frequently observed in pathological conditions such as mucositis [32]. Treatment with VER effectively normalized these serum markers, particularly ALP and LDH levels, suggesting its hepatoprotective properties. Notably, these effects were more pronounced at a low VER dose for ALP and a high VER dose for LDH. This may reflect VER's ability to promote tissue repair and regeneration, thereby preventing enzyme leakage. Our results are consistent with the previous studies demonstrating the ability of VER to lower serum enzyme levels [39–41].

5-FU is known to induce oxidative stress, which contributes significantly to its systemic side effects. Therefore, the serum MDA levels, which indicate lipid peroxidation and oxidative stress [42, 43], were measured to evaluate the effect of VER on 5-FU-induced gut injury. In this study, a significant increase in MDA levels was observed following 5-FU administration, indicating enhanced lipid peroxidation and oxidative stress, which can damage cellular membranes and other biomolecules. However, VER treatment effectively attenuated this 5-FU-induced increase in MDA levels. This suggests that VER possesses antioxidant properties, capable of scavenging free radicals and reducing lipid peroxidation, thereby mitigating oxidative stress caused by 5-FU. These findings align with previous studies demonstrating the antioxidant properties of VER [17, 18].

Chemotherapy-induced mucositis is exacerbated by increased levels of inflammatory cytokines such as IL-1β and TNF-α, which are important mediators of inflammation [4, 29, 44]. Consistent with these findings, administration of 5-FU in the present study increased serum levels of IL-1β and TNF-α. The elevated levels of these cytokines in 5-FU-treated rats indicate a strong inflammatory response that contributes to tissue damage. VER treatment significantly attenuated this cytokine elevation and concurrently reduced tissue damage. The significant reduction of these cytokines by VER may be attributed to its likelihood or ability to modulate signaling pathways involved in immune cell activation and the production of pro-inflammatory cytokines, particularly IL-1β and TNF-α. This attenuation of inflammation directly mitigates the inflammatory aspect of 5-FU-induced tissue injury. Our findings are consistent with previous reports on anti-inflammatory actions of VER in various disease models [14, 45, 46].

MMPs are proteolytic enzymes involved in various inflammatory diseases [47]. As released by inflammatory cells, MMPs degrade cellular debris during wound healing but lack specificity, leading to the destruction of essential extracellular matrix proteins [48]. Our findings demonstrate that 5-FU-induced gastrointestinal mucositis is associated with increased activation of MMP-1, -2, and -8. VER treatment effectively reduced serum levels of these MMPs, exhibiting a trend toward a dose-dependent effect in attenuating MMP-1 and MMP-8 expression. These findings align with previous studies on MMPs in diverse inflammatory contexts [46, 49].

MMPs and TIMPs maintain a balance that is essential for tissue remodeling [50]. it is known that 5-FU interferes with the signaling pathways that regulate gene transcription, which is involved in maintaining this balance. Dysregulation of MMP-TIMP balance can contribute to tissue damage. The current study observed an upregulation of MMPs and a concomitant downregulation of TIMP-1 in response to 5-FU-induced mucositis. Previous research on celiac disease and inflammatory bowel disease had reported similar imbalances, with elevated MMP-1 and MMP-2 and decreased TIMP-1 and -2 levels in the gut [51, 52]. TIMPs, especially TIMP-1 and TIMP-2, regulate MMP activity [53, 54]. Since treatment with VER significantly increased serum TIMP-1 levels, this may indicate a possible role in promoting tissue regeneration. The ability of VER to decrease MMP levels and increase the expression of TIMP-1 suggests a protective mechanism by maintaining the integrity of the extracellular matrix and reducing the hyperactivity of proteolytic enzymes that contribute to tissue damage in 5-FU-induced mucositis. Our findings are consistent with previous research highlighting the potential benefit of MMP inhibition in the treatment of mild to severe mucositis [55].

The 5-FU-induced decrease in albumin levels together with an increase in creatinine indicates a possible impairment of protein synthesis and possible damage to the liver or kidneys. Although treatment with VER did not significantly improve serum albumin or total protein levels, it effectively restored serum creatinine to levels close to control values. This suggests a possible protective effect of VER against some of the systemic toxicities caused by 5-FU. These results are consistent with previous reports on the influence of VER on serum biochemical markers [14, 56, 57].

Histopathological examination of the gastrointestinal tract in 5-FU-treated groups revealed severe tissue damage, characterized by marked degeneration, edema, and desquamation of the villi and glandular structures. These findings are consistent with previous reports describing desquamation, degeneration, and necrosis in the gastrointestinal tract following the administration of 5-FU [5, 58]. However, treatment with VER significantly alleviated tissue damage, promoted cell and tissue regeneration, and prevented necrosis. Overall, the histologic and biochemical results confirm that VER protects against 5-FU-induced gastrointestinal tissue injury.

## Conclusion

The protective effect of VER against gastrointestinal tissue damage was demonstrated by histologic improvements, decreased serum levels of hepatocellular enzymes (ALP and LDH), and decreased serum MDA levels. In addition, VER attenuated the 5-FU-induced increase in TNF-α, IL-1β, and MMP-1, -2, -8, key mediators of systemic inflammation. Based on these results, VER emerges as a promising adjuvant therapy to attenuate 5-fluorouracil-induced gastrointestinal mucositis in cancer patients. The present study exclusively utilized female albino Wistar rats. It is well known that sexual dimorphism can significantly influence the response to pharmacological interventions. Given the potential influence of genetic and physiological factors on these responses, both biological sexes should be used in future clinical and experimental studies to fully evaluate the cytoprotective effects of this agent against gastrointestinal mucositis.

## Data Availability

All data supporting the findings of this study are available within the article.
